# Sexual and Gender Minority Caregivers of People With Dementia and Their Care Recipients

**DOI:** 10.1093/geroni/igaa057.2443

**Published:** 2020-12-16

**Authors:** Joel Anderson, Jason Flatt, Jennifer Jabson Tree, Alden Gross, Karen Rose

**Affiliations:** 1 University of Tennessee-Knoxville, Knoxville, Tennessee, United States; 2 University of Nevada, Las Vegas, School of Public Health, Las Vegas, California, United States; 3 University of Tennessee, Knoxville, Tennessee, United States; 4 Johns Hopkins Bloomberg School of Public Health, Baltimore, Maryland, United States; 5 Ohio State University, Colombus, Ohio, United States

## Abstract

Little is known about the unique experiences of sexual and gender minority (SGM) caregivers of people with dementia or their care recipients. We used an electronic survey to assess psychosocial measures within this caregiving population, including measures related to the care recipient. The majority of caregivers (N=285) were gay men (62%). Most respondents were white (80%), with a quarter identifying as Latinx. The majority of caregivers were a spouse/partner (59.3%) and were providing care for someone who identified as LGB (70%), with 20% caring for someone transgender. Half of care recipients did not have an advance directive. The majority of care recipients needed assistance with ≥5 instrumental activities of daily living (83%) and ≥1basic activities of daily living (74%). This study is the first to provide data regarding the unique needs of SGM caregivers of someone with dementia and will support the development of targeted interventions for this population.

